# Extraction, Modification and Biomedical Application of Agarose Hydrogels: A Review

**DOI:** 10.3390/md21050299

**Published:** 2023-05-14

**Authors:** Feng Jiang, Xin-Wei Xu, Fu-Quan Chen, Hui-Fen Weng, Jun Chen, Yi Ru, Qiong Xiao, An-Feng Xiao

**Affiliations:** 1 College of Ocean Food and Biological Engineering, Jimei University, Xiamen 361021, China; 202011832019@jmu.edu.cn (F.J.); 202111832020@jmu.edu.cn (X.-W.X.); fqchenhy0109@jmu.edu.cn (F.-Q.C.); wenghuifen@jmu.edu.cn (H.-F.W.); chenjun@jmu.edu.cn (J.C.); 201611710043@jmu.edu.cn (Y.R.); 2 National R&D Center for Red Alga Processing Technology, Xiamen 361021, China; 3 Fujian Provincial Engineering Technology Research Center of Marine Functional Food, Xiamen 361021, China; 4 Xiamen Key Laboratory of Marine Functional Food, Xiamen 361021, China

**Keywords:** agarose, modification, hydrogel, biomedical application

## Abstract

Numerous compounds present in the ocean are contributing to the development of the biomedical field. Agarose, a polysaccharide derived from marine red algae, plays a vital role in biomedical applications because of its reversible temperature-sensitive gelling behavior, excellent mechanical properties, and high biological activity. Natural agarose hydrogel has a single structural composition that prevents it from adapting to complex biological environments. Therefore, agarose can be developed into different forms through physical, biological, and chemical modifications, enabling it to perform optimally in different environments. Agarose biomaterials are being increasingly used for isolation, purification, drug delivery, and tissue engineering, but most are still far from clinical approval. This review classifies and discusses the preparation, modification, and biomedical applications of agarose, focusing on its applications in isolation and purification, wound dressings, drug delivery, tissue engineering, and 3D printing. In addition, it attempts to address the opportunities and challenges associated with the future development of agarose-based biomaterials in the biomedical field. It should help to rationalize the selection of the most suitable functionalized agarose hydrogels for specific applications in the biomedical industry.

## 1. Introduction

Seaweed is an enormous marine flora and a major component of marine biological resources. About 90% of aquatic plant species are seaweed [1]. According to the composition of photosynthetic pigments, seaweed is usually divided into three groups: *Chlorophyceae* (green algae), *Phaeophyceae* (brown algae), and *Rhodophyceae* (red algae) [2]. Red algae, in particular, are the most diverse, containing around 6500 species [3]. The components of red algae, such as polysaccharides, proteins, and minerals, have been studied extensively. Polysaccharides have become the most exciting seaweed component for researchers because of their wide range of food and medicinal functions.

**Figure 1:**
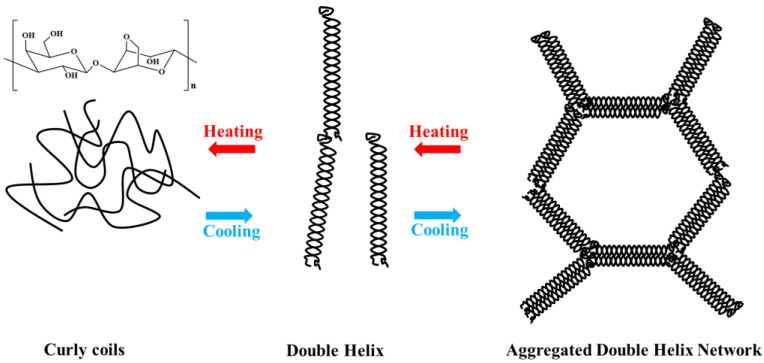
Structure and gelling mechanism of agarose [7].

Agarose, a natural polysaccharide obtained from red algae, is a linear polysaccharide consisting of d-galactose and 3,6-anhydrous-l-galactose with many hydroxyl groups in its structural unit [4], which readily form hydrogen bonds with hydrogen atoms in the structure or with those of water. Its molecular weight is generally between 80 kDa ~ and 140 kDa. Agarose can form a controlled hydrogel with stability and hysteresis [5]. As shown in Figure 1, at 90–100 °C, the hydrogen bonds between the structural units of agarose break, and agarose are dispersed into the water as random coils to form a clear solution. When the temperature is lowered to 30–40 °C, the molecular chains of agarose are intertwined by hydrogen bonds, forming a double helix structure that is tightly arranged to form a gel. According to recent research, the global agarose market looks promising for the next five years. The global agarose market was estimated to be USD 83.35 million by 2022 and is expected to reach USD 99.35 million by 2028, growing at a compound annual growth rate of 2.97% in the forecast years [6]. The end user segment of agarose is mainly in the biomedical sector, which includes academic institutions, hospitals, diagnostic centers, pharmaceuticals, and biotechnology. The regional features of this market include Asia Pacific, Europe, North America, the Middle East and Africa, and Latin America [6].

As functional biomolecules, bioactive polysaccharides are constantly being developed in the biomedical industry and are attracting considerable interest from scientists [8]. Polysaccharides and their derivatives from natural sources are preferred to synthetic substances in food, household products, and pharmaceuticals because of their biodegradability, non-toxicity, and biocompatibility [9,10,11]. Polysaccharides are now widely used for disease diagnosis, inhibition and treatment, drug delivery, antibacterial and antiviral applications, and tissue engineering [9,12,13,14]. Agarose is a natural source of neutral gel polysaccharide, which plays a vital role in the biomedical industry. It can inhibit the growth of bacteria on the surfaces of medical devices because of its low viscosity as an antimicrobial material [15,16]. The excellent biocompatibility of agarose makes it suitable as a hydrogel for the controlled release of drugs [17,18] and materials for tissue engineering [19,20]. Agarose gel is electrically neutral and rigid; therefore, it is widely used as a filling material for gel electrophoresis and separation media. Agarose can be modified by compounding or grafting with other materials to provide specific adhesion and a pH response [17], making agarose more promising for tissue regeneration and other applications.

In the present work, the main emphasis has been placed on applying agarose and its derivatives in the biomedical field. Firstly, an overview of agarose extraction is presented. Next, the preparation of agarose derivatives is summarized, and the principles, advantages, and disadvantages of the modification methods are briefly discussed. A comprehensive review of agarose applications in the biomedical field is then presented. This review considers four categories: agarose gel electrophoresis, agarose separation media, agarose coatings, agarose as a drug delivery vehicle, and agarose-based tissue engineering materials.

## 2. Extraction and Modification of Agarose

### 2.1. Agarose Extraction

In 1937, Araki first isolated agarose from agar by acetylation, and then scientists began to study the extraction of agarose [21]. Agar is generally extracted from red algae, and electrically neutral agarose can be obtained by removing the agar proteins [22]. At present, many studies on agarose extraction techniques have been reported [22,23]. According to the extraction principle, it can be classified into agaropectin precipitation, agarose precipitation, ion exchange, ionic liquid, and complex extraction. Table 1 shows the extraction methods for agarose.

#### 2.1.1. Agaropectin Precipitation Method

Agar generally consists of agarose and agaropectin, and agarose and agaropectin dissolve at different rates in different solvents. Therefore, the researchers used a specific solvent to dissolve the agar and separate the insoluble portion; the part dissolved in the solvent was agarose. Jeon et al. [23] isolated agarose with a low sulfate content and high strength from agar by precipitating agaropectin-containing sulfate using DMSO. Santos et al. mixed quaternary ammonium compounds with an agar solution to form a precipitate and dried the filtrate to obtain agarose. To solve the problem of the difficult separation of agarose during the extraction of agarose by the quaternary method, Blethen et al. [24] used λ-carrageenan together with agarose to form an easily separable precipitate, which could be quickly separated by quaternary ammonium salts.

#### 2.1.2. Agarose Precipitation Method

The agarose precipitation method is also based on the difference in solubility of agarose and agaropectin in the solvent. The difference is that the precipitated part is agarose. The most commonly used method is the polyethylene glycol method [22]. Duckworth et al. [25] added polyethylene glycol to the agar solution, dissolved in 0.05 M NaCl at 50–60 °C, stirred continuously to produce a precipitate, collected the precipitate after centrifugation, washed the precipitate with polyethylene glycol and NaCl solution, and dried to obtain agarose. Chew et al. [22] dissolved polyethylene glycol in ethanol at 70–75 °C, mixed the same amount of agar solution with a polyethylene glycol-ethanol solution, stirred, produced a white flocculent precipitate, and centrifuged. The precipitate was washed with distilled water and acetone, freeze-dried, and ground to obtain agarose.

#### 2.1.3. Ion Exchange Method

Ion exchange is the ion exchange process between ions in a solution and ions in an ion exchange resin to remove specific ions from the solution [26]. The anion exchange resin method is commonly used to extract agarose [27]. Anion exchange resins adsorb negatively charged anions from the solution and can be used to separate agarose from agar. Duckworth et al. [25] added an agar solution treated with polyethylene glycol to DEAE-Sephadex A-50, eluted with distilled water, and then added the eluate to ethanol and precipitated to obtain agarose. Zhang et al. [28] added an agar solution to DEAE-cellulose suspension and stirred at 70 °C for two hours; agarose was prepared by freezing, dehydration, and drying.

#### 2.1.4. Ionic Liquid Method

Ionic liquids (ILs) are widely used to extract various chemicals or biopolymers from biomass because of their high ability to dissolve polar and non-polar compounds [29]. In addition, ILs can be effectively recovered and recycled. In recent years, ILs have also been used as a novel solvent for agarose extraction. With constant stirring, Sharma et al. [30] added the ILs solution to the alkali-treated seaweed extract, and the resulting precipitate was washed with gradient IPA and vacuum-dried agarose products. Trivedi et al. [29] mixed seaweed powder with a preheated ILs solution, heated and filtered after 2–3 s of microwave treatment, and the filtrate was precipitated with methanol. The precipitate was collected and removed with a methanol solution; purified agarose was obtained by vacuum drying after removing residual ILs with a methanol solution.

**Table 1:** Extraction methods for agarose.

Method	Reagents	Seaweed Type	Highlights	Cite
Agaropectin precipitation	DMSO	*Gelidium amansii*	1 wt% concentration gel strength was 1190 g/cm^2^; sulfate content was 0.28 wt%	Jeon et al. [23]
	Quaternary ammonium compounds	*Gracilaria cylindrica*	1 wt% concentration gel strength was 935 g/cm^2^; sulfate content was 0.17 wt%	Santos et al. [24]
		*Gelidium amansii*	1 wt% concentration gel strength was 742 g/cm^2^; sulfate content was 0.63 wt%	Chew et al. [22]
Ion exchange	DEAE-Sephadex A-50	Purified agar solution	sulfate content was 0.05 wt%; pyruvic acid content was below 0.01 wt%	Duckworth et al. [25]
	DEAE-Cellulose suspension	*Ahnfeltia plicata*	1 wt% concentration gel strength was 1417 g/cm^2^; sulfate content was 0.15 wt%	Zhang et al. [28]
Ionic liquid	Choline-based bio-ionic liquids	*Gracilaria dura*	1 wt% concentration gel strength was 1250 g/cm^2^; sulfate content was 0.21 wt%	Sharma et al. [30]
	1-ethyl-3-methylimidazolium acetate, choline acetate, 1-ethyl-3-methylimidazolium diethyl phosphate	*Gracilaria dura*	1 wt% concentration gel strength was 600 g/cm^2^; sulfate content was 1.95 wt%; yield was 39 wt%	Trivedi et al. [29]
	Heat-compatible; strong-anion exchange; isopropanol	*Gracilaria amansii*	1 wt% concentration gel strength was 853 g/cm^2^; sulfate content was 0.14 wt%	Wang et al. [31]

#### 2.1.5. Agarose Extraction by a Complex Method

To improve the purity of agarose, researchers have attempted to combine different techniques to extract agarose. Zhang et al. [28] used a mixture of polyethylene glycol and anion exchange resin to remove agaropectin. Agarose was dissolved after extraction with polyethylene glycol and further purified by anion exchange resin. Wang et al. [31] preliminarily separated agarose and impurities by heat-compatible anion exchange resin after treating the agar with EDTA and then dissolved agarose before adding isopropanol for alcohol precipitation, collecting the precipitate and freeze-drying and purifying to obtain biotechnological grade agarose. This method effectively reduced the sulfate content of agarose. 

Nowadays, with the increasing awareness of environmental protection, researchers are constantly improving the extraction of agarose to develop a simple, fast, efficient, economical, and environmentally friendly method. In recent studies, researchers have found that enzymatic desulphurization efficiently obtains agarose [32]. The DNA electrophoresis profiles of the agarose gel obtained were indistinguishable from those of commercially available agarose [33,34]. In addition, researchers have found that H_2_O_2_ can effectively remove the C-4 sulfate group of d-galactose from the agar and that the H_2_O_2_ decomposition products are non-polluting; therefore, the H_2_O_2_ method is expected to be a new agarose production method [35,36].

### 2.2. Modification of Agarose

Agarose gel exhibits brittleness and contractility, which makes it problematic in applications such as tissue engineering and wound dressings. To improve these shortcomings, biological, physical, and chemical modification methods were used to obtain a good performance by changing the molecular structure of agarose [37]. Table 2 shows the modification methods for agarose.

Biological modification, typically using enzymatic modifications, such as reducing the sulfate content by sulfatase [38], is a green, gentle, and efficient method of modifying polysaccharides. A physical modification is the alteration of the original morphology and structure of polysaccharides with physical techniques such as micronization, ultrasound, high-pressure homogenization, moist heat treatment, and pulsed electric fields [39]. It alters the original properties of the polysaccharide by affecting physical interactions, including hydrogen bonding, electrostatic interactions, hydrophobic interactions, or intermolecular interactions [40], Krömmelbein et al. [41] treated agarose hydrogel with electron irradiation. They found that the glycosidic bonds of agarose hydrogel were weakened, and carbonyl-containing substances were formed during electron irradiation treatment. Chemical modification aims to add new groups to a polysaccharide and provide it with unique properties [39]. Sulphation, carboxymethylation, acetylation, and phosphorylation are the main methods for the chemical modification of polysaccharides. The chemical modification of polysaccharides leads to structural diversity and helps to create new gel properties. In general, biological modifications are costly and require strict reaction conditions, while physical modifications do not increase polysaccharides’ biological activity and usually result in reduced quality [40]. By contrast, chemical modifications are preferred by researchers due to their simplicity, high efficiency, and reproducibility.

At present, low-melting agarose is usually prepared by chemical modification. Zhang et al. [42] showed that the low-melting agarose designed by oxy alkylation has good thermal reversibility, mechanical properties, and separation efficiency and is also a promising and economical low-melting agarose; it is the most common low-melting agarose on the market. Xiao et al. [43] successfully modified agarose with octenyl succinic anhydride (OSA), which showed new gel properties such as low gel strength, low melting temperature, and high transparency compared to the native agarose. Furthermore, Xiao et al. [44] prepared agarose-fatty acid esters using fatty acid derivatives. They found that the preferred substitution site was the hydroxyl group at the C-2 position of D-galactose. They also found that long-chain fatty acids and highly substituted agarose had a higher emulsifying capacity than short-chain fatty acids and low-substituted derivatives. Oza et al. [45] prepared a new fluorescent polymeric material by grafting the nucleobase guanine onto agarose using a water-based method and potassium persulfate as an initiator. These studies suggest that the properties of introduced side chains can predict new properties of modified products, which also provides ideas for the development of other agarose derivatives.

**Table 2:** Modification of agarose.

Method	Type	Application	Modification Reagents	Results	Cite
Biomodification	Enzymatic modification	Edible packaging film	Galactose oxidase GAO-5F	Agarose is oxidized to polyaldehydes and can be cross-linked with gelatine for application in food packaging films.	Cao et al. [46]
	Enzymatic modification		GH50 Agarosease Aga3420	Efficient production of high purity neoagarobiose (NA2) in a low temperature environment.	Zhang et al. [47]
Physical modification	Electronic irradiation			Material properties of agarose hydrogels can be adjusted at a low doses of high energy electron irradiation.	Krömmelbein et al. [41]
	Ultrasound	Drug delivery	1-MHz Ultrasound	High frequency ultrasound sonication enhances the internal diffusivity of agarose gels and aid drug delivery.	Tsukamoto et al. [48]
	Compounding		Mucin	Crosslinking between mucin and agarose results in increased swelling, adhesion, hygroscopicity, and thermal properties.	Builders et al. [49]
	Compounding	Drug delivery	Chitosan/γ-alumina	Hydrogel nanocomposites are efficient drug delivery systems for the chemotherapeutic agent 5-FU and simultaneously reduce its adverse effects.	Bayat et al. [50]
	Compounding	Drug delivery	Fe_3_O_4_/CS	Ability to release curcumin in response to pH	Pourmadadi et al. [51]
Chemical modification	Esterification		Octenylsuccinic anhydride	Reduced gel strength, lower melting temperature and increased transparency	Xiao et al. [43]
	Esterification	Emulsifiers	Fatty acid derivatives	Improved emulsification properties	Xiao et al. [44]
		Sensor	Nucleobase guanine	Good fluorescent activity	Oza et al. [45]
	Esterification	Drug delivery	Carbonyl diimidazole	Adsorbs more hydrophobic dyes and controls the release of hydrophobic dyes	Evans et al. [52]
	Esterification	Microcapsules	Dodecenylsuccinic anhydride	Prepared agarose microcapsules were used for the encapsulation of DHA and showed good oxidative stability and release properties.	Xiao et al. [53]
	Conjugated	Immunoaffinity chromatography columns	Avermectin polyclonal antibodies	Enables the rapid and sensitive simultaneous determination of avermectin, ivermectin, doramectin, and eprinomectin residues in the bovine liver and muscle in combination with LC-MA-MA	Hou et al. [54]
	Coupling	Hydrophobic interaction chromatography support	Phenyl ligand	Microspheres can be used to isolate lysozyme and bovine serum proteins and can tolerate higher flow rates.	Gustavsson et al. [55]

## 3. Biomedical Applications of Agarose and Its Derivatives

**Figure 2:**
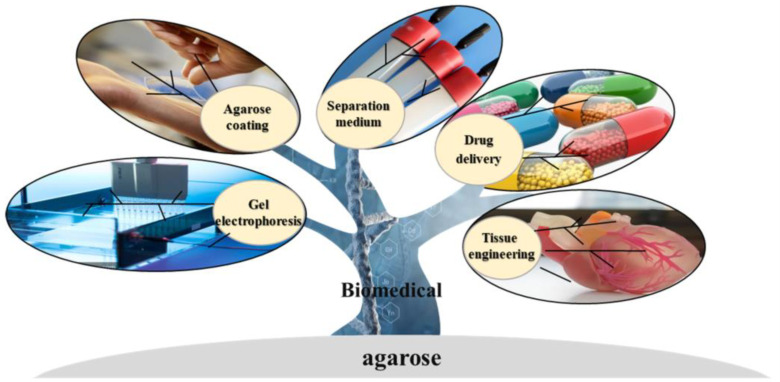
Application of agarose.

As the sol—agarose chains that form a porous network through hydrogen bonding [43]—are gelled, it allows the diffusion of high molecular weight substances. Meanwhile, many biological processes, such as the separation and purification of biological macromolecules, the complexation of proteins, and cell growth and multiplication, depend on buffers. For this reason, the hydrophilicity of the medium is essential for use in biological processes. Agarose is a polyhydroxy hydrocolloid, and the diffusion of biomolecules in the gel is not clearly different from that in an aqueous solution [56]. Synthetic and inorganic biomaterials, such as polyacrylamide gel and porous glass, are limited in separation and purification because of their nonspecific adsorption with some biomacromolecules. In theory, biologically active substances interact weakly with agarose and can remain biologically active in agarose gel [57]. It is possible to functionalize agarose to obtain specific properties that can lead to the derivation of different types of chromatographic separation media, extending the range of separation applications [58]. Figure 2 shows the application of agarose and its derivatives in the biomedical field.

### 3.1. Agarose Gel Electrophoresis

Electrophoresis is increasingly used in several fields, including clinical chemistry, toxicology, pharmacology, and immunology. Electrically charged molecules are driven through the agarose matrix by an electric field. They are separated in the agarose gel matrix according to their size, conformation, shape, and amount of charge they carry [59]. For this reason, agarose gel electrophoresis is widely used to separate and identify biological macromolecules such as nucleic acids, polysaccharides, proteins, and viruses [60,61].

The concentration of agarose gel electrophoresis is usually in the range of 0.5 to 2% (*w*/*w*) [62,63]. Agarose gel electrophoresis has the advantage of easy operation, simple equipment, a low sample volume, and high resolution. It serves the dual purpose of a “molecular sieve” and “electrophoresis” and is widely used in the study of nucleic acids [59]. In particular, chemically modified agarose with a low melting point has a higher sieving capacity and is ideal for the electrophoresis of DNA and RNA, allowing the recovery of the natural forms of DNA from the gel [64]. Green et al. [65] separated DNA fragments of different sizes using low-melting agarose and then recovered DNA by organic extraction from molten agarose using phenol-chloroform.

Zhang et al. prepared low melting point agarose through the oxy alkylation of agarose using ethylene oxide, 1,2-epoxypropane, and 1,2-epoxy butane [42]. They found that oxy-alkylated agarose underwent a superior separation performance in DNA gel electrophoresis. Recently, cyclic RNA migration under various conditions, various monoclonal and polyclonal antibodies, and the molecular weight of serum albumin have also been shown to be analyzed by agarose gel electrophoresis. Li et al. [62] characterized monoclonal antibodies developed against short peptide phosphotyrosines for reactive kinase phosphorylation by agarose gel electrophoresis. They optimized electrophoretic conditions by adjusting critical parameters of the electrophoretic process. Tomioka et al. [66] performed the agarose gel electrophoresis of commercially available bovine serum proteins using UltraPure and MetaPhor agarose. They found that the agarose gel electrophoresis obtained with MetaPhor agarose had a more significant molecular sieving effect, with high-resolution size differences and the ability to show more than 4%, oligomeric bands. In addition, agarose gel electrophoresis has been used to separate proteins and their complexes. Sakuma and colleagues [67] effectively extended the scope of agarose gel electrophoresis for biomedical applications by using agarose gel electrophoresis to analyze physically modified, aggregated, or post-translationally modified proteins. Agarose can also be mixed with polyacrylamide and dextran to support gel electrophoresis.

### 3.2. Agarose Separation Medium

Agarose gel is a hydrophilic medium that is highly compatible with biological macromolecules. Its porous properties make it ideal for separating proteins and nucleic acids [68]. Additionally, agarose can be customized for various applications by incorporating other functional groups as needed [69]. Agarose is a popular chromatographic separation media due to its availability in various forms, such as affinity, ion exchange, and hydrophobic chromatography media [70]. These different forms are based on distinct separation principles, making agarose one of the most widely used polysaccharide-based separation media.

#### 3.2.1. Affinity Chromatography

Affinity chromatography is a technique used to separate and purify biological macromolecules by taking advantage of their specific recognition or reversible binding to corresponding molecules [71]. Agarose is a preferred matrix for affinity chromatography due to its low cost, large pore size, minimal non-specific binding to biological reagents, and stability across a wide range of pH values [72,73]. Affinity chromatography can be classified into four main types based on the interaction systems between biological macromolecules and ligands. These types are affinity chromatography, biomimetic affinity chromatography, immunoaffinity chromatography, and metal ion affinity chromatography.

Biological affinity chromatography is a kind of affinity chromatography that uses highly selective biological specificity to select interacting substances [74]. The enzyme-substrate, enzyme-inhibitor, and hormone-receptor pairs are commonly used. Yi et al. [75] exploited the biospecific choice between enzymes and enzyme inhibitors by using ST/SC (SpyTag/SpyCatcher) chemistry and targeted the immobilization of the green fluorescent protein as a model protein on agarose microspheres. Subsequently, ST-GFP (green fluorescent protein) was replaced by ST-PqsA (a key enzyme in the *Pseudomonas aeruginosa* quorum sensing pathway), and PqsA inhibitors were isolated by a biospecific selection between PqsA and PqsA inhibitors. Biomimetic affinity chromatography uses molecular interactions to synthesize ligands that mimic specific structures and sites on biological molecules as stationary phases for protein adsorption. Bai et al. [76] isolated and purified formate dehydrogenase (FDH) from bovine cell extracts by immobilizing four triazine reactive dyes on agarose and found that Procion Blue HERB was a suitable dye ligand for FDH, enabling simple, inexpensive, and efficient agarose affinity chromatography. Immunoaffinity chromatography is a separation system in which one of the antigens and antibodies is used as a ligand to affinity adsorb the other side. Hou et al. [54] prepared immunoaffinity chromatography columns to separate avermectin, ivermectin, doramectin, and ivermectin from samples by coupling avermectin polyclonal antibodies to CNBr-activated agarose, followed by an analysis of the residues eluting from the columns by high-performance liquid chromatography-tandem mass spectrometry. Metal ion affinity chromatography uses metal ion complexes to bind to proteins for separation and purification. The polymer, supported by super-porous agarose particles, has a high osmotic pressure, a high tolerance to interference mechanisms, and low back pressure. Zheng et al. [77] constructed complex immobilized metal ion affinity agarose particles to selectively isolate and purify histidine-tagged proteins using super porous agarose particles as supports, flexible copolymer brushes as backbones, and Ni^2+^-chelated iminodiacetic acid as ligands.

#### 3.2.2. Size Exclusion Chromatography

Size exclusion chromatography (SEC) is a chromatographic technique for the separation of polymer samples based on the correspondence between the pore size of the gel pores and the molecular size of the polymer, with the advantages of the simplicity of the operation and high sample recovery [78]. Because of the 3D network structure of the gel filtration media, biomacromolecules with high molecular weight are blocked on the outside as they pass through the pores of gel particles and elute directly downwards at a rapid rate. By contrast, biomacromolecules with a low molecular weight can enter the interior of the gel particles as they pass through the column and are retained at a slow elution rate. The molecular sieving effect of the gels allows the molecular size of the samples to be screened for different elution times for biomolecules of different molecular weights [79]. Dextran, polyacrylamide, and agarose gel are the most commonly used gel filtration media. In particular, agarose gels have a higher mechanical strength and sieve stability, allowing for higher flow rates, a fuller range of pH conditions, and molecular weights. Size exclusion chromatography is often divided into gel permeation chromatography and gel filtration chromatography, depending on the mobile phase.

Zhao et al. [80] prepared homogeneous agarose microspheres of controlled particle size by an emulsion membrane method, followed by multi-step cross-linking and dextran grafting to obtain high-resolution chromatography media, finely controlling the molecular range with good pressure resistance. Site-specific glycosylation and its associated heterogeneity affect the functional activity of glycoproteins, and studies have shown that aberrant glycosylation can be closely associated with many diseases. Yet, the analysis of intact glycopeptides remains a significant challenge. For this purpose, Zhao et al. [81] developed a method to enrich intact tryptic N-glycopeptides using the excellent properties of the acrylamide–agarose composite gel and the hydrophilic properties of volume exclusion chromatography.

#### 3.2.3. Ion Exchange Chromatography

Ion exchange chromatography (IEC) is a separation method based on the difference in electrostatic interactions between the sample and the stationary phase (ion exchange chromatography medium [82,83]). It has the advantages of wide applicability, high resolution, no effect on the biological activity of the sample, and high resolution, and it is widely used for the separation and purification of biological macromolecules [84] such as proteins [85]. The derivatizability of the hydroxyl groups of agarose allows ligands of different charge types and densities to be introduced into the gel [82]. Depending on the properties of ion-exchange ligands coupled to the agarose surface, IEC can be divided into anion-exchange chromatography media and cation-exchange chromatography. Currently, the most frequently used functional ligands are diethyl aminoethyl [86], carboxymethyl [87], and sulfopropyl [88], which make an agarose-based ion-exchange medium for weak anions, strong anions, weak cations, and strong cations, respectively. Separation can be achieved according to the difference in electrostatic interactions between the separation medium and the molecules because of the difference in charge type, charge number, and charge distribution on the surface of different molecules. In specialty biochemical separation media, the Agarose range of ion exchange media is currently a mainstream product. Anion-exchange chromatography separates and purifies positively charged proteins (lysozyme and cytochrome C) using an agarose-based anion-exchange medium. Silva-Santos et al. [89] used anion-exchange chromatography with HiTrap Q-agarose as the anion-exchange medium to purify ssDNA generated by aPCR from the reaction mixture. Barroca-Ferreira et al. [90] used Q-Sepharose anion-exchange chromatography to efficiently and finely purify prostaglandin 1.0 from K. pastoris small bioreactor lysates in a series of separation and purification steps; this is involved in cellular communication, stimulating cell proliferation and is specific to the cancer microenvironment. Cation exchange chromatography combines an ion exchange stationary phase with a negatively charged moiety coupled to a positively charged cation for separation and purification. Li et al. [91] investigated the effect of chain length and ionic strength on lysozyme adsorption and chromatographic interactions with γ-globulin by developing a cation exchange medium and co-grafting sodium methacrylate onto the commercial agarose gel Sepharose FF. Zhao et al. [70] developed a method by combining pre-crosslinking and surfactant micelle swelling to produce highly crosslinked macroporous agarose microspheres with a homogeneous network structure, low backpressure, and high flow rate, and, based on this, they created a carboxymethyl-coupled cation exchange chromatography media that could effectively separate proteins at high flow rates. Using sulphopropyl-Sepharose cation exchange chromatography, Li et al. [92] isolated and purified a 17.5 kDa bean protein inhibitor with anti-proliferative activity against leukemia and lymphoma cells from canola.

#### 3.2.4. Hydrophobic Interaction Chromatography

Hydrophobic interaction chromatography (HIC) is a method that uses the reversible binding of a hydrophobic target in the mobile phase to the separation media together with a hydrophobic binding partner to achieve separation and purification [93]. HIC is widely used for the isolation and purification of biomolecules, such as proteins and peptides [55,93,94], for the advantages of mild reaction conditions, the high recovery of biomolecules, a low environmental impact, and cost savings [95,96]. They are typically composed of a hydrophobic matrix with a hydrophobic ligand. Currently, HIC media are commonly used with natural polysaccharides such as agarose and cellulose and synthetic polymers such as polystyrene and polyacrylates [97]. 

Agarose hydrophobic chromatography media consisting of agarose and hydrophobic ligands were introduced by chemical modification. Here, there are two main types of hydrophobic ligands: a hydrocarbon group with different carbon chain lengths, CH_3_(CH_2_)_n_-X, where X can be NH_2_, COOH, or OH, and hydrophobicity increases with the value of n; the second is a butyl or phenyl group with general hydrophobicity [98,99]. Its separation is based solely on the strength of the hydrophobic interaction, as the absence of groups such as NH_2_, COOH, or OH eliminates the interference of hydrogen bonding or charge interactions.

Superporous agarose beads are frequently employed as a foundation for hydrophobic chromatographic media. Gustavsson et al. [55] conducted a study in which they separated ribonuclease A, lysozyme, and bovine serum albumin using hydrophobic agarose chromatographic media prepared from phenyl-derived super porous agarose beads. The results showed that these proteins could be effectively separated and purified. The use of the chromatographic method resulted in higher flow rates compared to the use of homogeneous agarose beads as support. Hydrophobic chromatography was found to provide even higher flow rates. Lipases play a crucial role in various fields, such as oleochemistry, organic chemistry, biofuels, and pharmaceuticals, due to their ability to catalyze the hydrolysis of triacylglycerols into glycerol and free fatty acids. Mehta et al. [100] purified lipases from Aspergillus fumigatus through the use of octyl-Sepharose column chromatography for this purpose. The activation of angiogenesis by an essential fibroblast growth factor (bFGF) and vascular endothelial growth factor A (VEGFA) could promote tumorigenesis. However, the use of peptidomes containing bFGF/VEGFA can effectively block the activation of these growth factors, making them a promising option for the development of therapeutic tumor vaccines. Holková et al. [101] employed phenyl agarose CL-4B hydrophobic chromatography to isolate and purify lipoxygenases from poppy cultures. These enzymes play a crucial role in regulating growth, development, and resistance to biotic and abiotic stresses.

### 3.3. Agarose Coating

Infections caused by various bacterial biofilms have become one of the most common public health problems in clinical settings [102]. Bacterial proliferation and perithecia formation on medical devices pose a serious health challenge to patients [15]. As a result, the provision of anti-fouling and anti-bacterial capabilities for medical devices has become a hot research topic [103]. One of the most effective ways of doing this is to form coatings of a certain thickness and adhesion on the surface of the equipment. Agarose and its derivatives have been widely used as substrates for functional hydrogel coatings due to their excellent lubricity, biocompatibility, electrical conductivity, and mechanical properties [102,104]. 

Agarose shows no excellent anti-fouling or anti-bacterial properties. Early research has shown that agarose gel could resist the attachment of *A. aeruginosa* at a pH below 8.0, but when the pH increased beyond 8.2, the bacteria aggregated and was deposited on the agarose surface. Therefore, the majority of research has been carried out to produce novel coatings by combining agarose with other substances, commonly combining materials with antimicrobial properties and agarose [105]. Eric et al. [106] developed a novel coating by incorporating Cu/bioactive glass into an agarose matrix and showed that released Cu improved anti-adhesive properties and slowed biofilm formation on the biomaterial surface. Li et al. [107] combined ZnO nanoparticles and Ag nanoparticles on silk glue–agarose composites, which showed excellent antibacterial activity against Gram-positive and Gram-negative bacteria and great potential as a novel antibacterial biomaterial. Rather than simply compounding the agarose, chemical reactions such as crosslinking and grafting bound the agarose to different molecules of different sizes. This method improved the antibacterial and contamination resistance of agarose while maintaining superior gelling properties. Li and colleagues [103] introduced acrylate groups onto agarose molecules to form a covalent, crosslinked agarose coating on the surface of a medical silicone diaphragm that reduced the formation of bacteria by more than two orders of magnitude. He grafted and cross-linked agarose and quaternary ammonium chitosan using thiol-alcohol chemistry as an antimicrobial coating. This crosslinked coating effectively inhibited the biofilm formation of Gram-negative and Gram-positive bacteria. Interestingly, after 30 days of repeated wiping with ethanol, autoclave, or lysozyme solutions, the layer retained its antimicrobial activity [108].

### 3.4. Drug Delivery

A wide variety of drug carriers have been developed and are in use. Still, drug delivery has been on a path of innovation due to multidimensional challenges from materials to cost [109]. Carbohydrate polymers are one of the most important materials for the design of efficient drug carriers and have great potential to meet a wide range of needs [110,111]. Common carbohydrate polymers such as chitosan, starch, and cellulose have been widely used in drug delivery systems but still have considerable drawbacks. For example, natural chitosan is only soluble in dilute acidic aqueous media and loses its mechanical properties [112]; starch has poor mechanical properties and high-water solubility [113]; cellulose is insoluble in organic and aqueous solvents and has poor antibacterial properties [114]. All of these are unavoidable shortcomings. Agarose, with its non-toxicity, gelatinization, and gel structure with suitable viscoelasticity and thermal reversibility, is a common substrate for constructing particles, microcapsules [115], and microspheres and is excellent for controlled drug release. Drug release systems can be built using the biocompatibility and haemocompatibility of agarose [116]. For example, active ingredients can be encapsulated in agarose hydrogel nanoparticles and prepared by the emulsion template method [117]. Agarose can also be mixed with various polysaccharides (e.g., chitosan, cyclodextrin, and konjac glucomannan), proteins, and magnetic nanoparticles to form complexes with enhanced physicochemical properties for efficient drug delivery [116]. In general, agarose hydrogel drug delivery systems are dominated by mass diffusion release (Fickian diffusion) in agarose hydrogels, where the diffusion time depends on the network structure and the size of the particles [118]. In targeted drug delivery systems, agarose gels can be adapted in terms of their pore size, structure, and function by adjusting the agarose concentration [119], mixing with other compounds, and making chemical modifications to create an interesting and versatile drug delivery system [118].

In 1994, Haglund et al. [120] loaded Ibuprofen and Indomethacin onto agarose beads and studied their release mechanisms. To reduce the severe side effects associated with high doses of cyclophosphamide as a premedication in oncological treatment, Sakai et al. [121] encapsulated cells that were genetically modified to express cytochrome P450 2B1 enzymes in sub-sieve-sized agarose capsules before placing the cell-encapsulated microcapsules in arteries near the tumor via a microcatheter and activating them locally. The side effects were effectively reduced without reducing the dose. The subcapsules of agarose were smaller in diameter than standard agarose, reducing surgical trauma while also reducing the occlusion of the dripping vessel.

Apart from agarose pellets for drug delivery, agarose hydrogels are widely used in drug delivery systems due to their biocompatibility and solute permeability [122]. However, natural agarose has a high mechanical strength and solidification temperature [42], making it difficult to load thermosensitive drugs due to their inactivation during natural agarose gelation. As mentioned above, Kim et al. [42] prepared low gel temperature agarose by introducing β-cyclodextrin into ethylenediamine-modified agarose and found that this gel could be used for the sustained release of the thermosensitive drug doxorubicin because of its low mechanical strength and solidification temperature. In addition, hydrogel complexes of agarose with polysaccharides and proteins are often used as carriers for drug delivery systems to compensate for the shortcomings of natural agarose hydrogel in drug delivery. Rossi et al. [123] used agarose in a synthetic hydrogel with a Carbomer 974P macromolecule monomer as a spatially blocked and molecularly structured drug and mimetic molecule sodium fluorescein as a drug delivery vehicle.

To overcome the limitations of conventional therapeutics and increase the relevance and sensitivity of the drug delivery process, materials that respond to a range of endogenous (pH, enzyme expression, and redox potential) or exogenous (temperature, ultrasound, magnetic fields, and light) stimuli can be used as substrates for drug delivery systems [124]. Agarose hydrogel is electrically conductive, pH-responsive, and thermally reversible, making it an ideal tunable drug delivery system for loading and controlling the release of drugs by stimulating a sensitive response under different environmental conditions, increasing the retention time of the drug, and thus, reducing the dose frequency and toxic effects of the drug [125]. Endogenous drug delivery directly transfers a carrier-bound drug to a target site. Rajabzadeh-Khosroshahi et al. [126] prepared a degradable and biocompatible chitosan/agarose/graphitic carbon nitride nanocomposite for the loading and delivery of anti-cancer curcumin. It was found that the nanocomposite particles were effective at enhancing the bioavailability of curcumin in pH-sensitive drug release studies. Pourmadadi et al. [127] developed an agarose/chitosan double nanoemulsion as a drug delivery vehicle for curcumin/5-fluorouracil using a green synthesis approach. This nanocomposite was highly efficient for delivering curcumin/5-fluorouracil and has excellent potential for the targeted treatment of cancer cells. Quercetin, a drug with anticancer properties, is limited in cancer treatment due to its low solubility, low permeability, and short half-life. To this end, Samadi et al. [128] loaded quercetin into a pH-responsive agarose-polyvinylpyrrolidone-hydroxyapatite hydrogel nanocomposite and encapsulated it in the internal aqueous phase of a w/o/w emulsion and found that it was effective at enhancing the loading capacity, sustained release, and apoptosis-inducing effects of quercetin. Exogenous drug delivery is the release of a drug from a carrier to a target site that is triggered by various stimuli. Dong et al. [129] developed a drug-loaded MXene/agarose hydrogel drug delivery system with high photothermal conversion efficiency and photothermal stability by loading MXene nanosheets together with the anticancer drug adriamycin into a low-melting point agarose hydrogel, allowing for controlled drug release by near-infrared light irradiation. Hu et al. [130] prepared a pH- and magnetic field-responsive drug delivery system for the administration of adriamycin hydrochloride by grafting and copolymerizing 2-hydroxyethyl acrylate and vinyl acetate Fe_3_O_4_@agarose nanoparticles for the efficient and sustained release of adriamycin hydrochloride in the presence of weak acid or external magnetic fields.

### 3.5. Tissue Engineering

Tissue engineering combines tissue cells with specific biological activity and biomaterials to construct tissues and organs in vitro or in vivo to maintain, repair, regenerate or improve the function of damaged tissues and organs. The scaffold material serves as an artificial extracellular matrix for tissue engineering [131]. It supports cell attachment, growth, reproduction, metabolism, and the formation of new tissues. Because of the unique properties of human tissues, determining the best type of scaffold remains challenging. The most researched materials are biodegradable polymers, ceramic-like materials, composite materials, and materials derived from biological sources [132]. Natural carbohydrate polymers are crucial in tissue engineering scaffolds because they are non-toxic and biocompatible [133]. Agarose has unique electrical neutrality and gelling properties compared to other common carbohydrates such as chitosan and alginate [134]. There are a wide variety of agarose-based tissue scaffolds, including hydrogel, 3D/4D printed scaffolds, etc.

#### 3.5.1. Agarose Hydrogel

Hydrogel, with its ability to provide a microenvironment for cell adhesion, proliferation, and migration, and facilitate biomolecular exchange, is essential to tissue engineering. Agarose has excellent potential for various tissue engineering applications because of its controlled self-assembly properties and tunable water adsorption capacity [133,135]. The structure, composition, and mechanical properties of single agarose are not conducive to customization and are usually complex with other polysaccharides to extend its range of applications [134]. Su et al. [136] prepared the agarose polydopamine (APG) hydrogel as scaffolds for skin wound healing. APG has good cell adhesion and high cell migration on the surface of the hydrogel, allowing cells to penetrate the hydrogel network. This study also showed that a composite hydrogel accelerated the healing process of full-thickness skin defects. In addition, the hydrogel can be used for cartilage tissue engineering. Singh et al. [134] prepared mulberry and non-mulberry silk hydrogel mixed with agarose and evaluated cartilage tissue formation in vitro. The results showed that mixed hydrogel exhibited enhanced cell proliferation and collagen deposition from sulfated glycosaminoglycans, clearly indicating that the composite hydrogel is a potential alternative for cartilage repair.

#### 3.5.2. 3D/4D Printed Brackets

Three-dimensional printing uses computer-aided design models to produce solid objects in a precise layer-by-layer deposition [137], enabling biological structures that mimic the structure and functional properties of in vivo tissues and are of great importance in the field of tissue engineering [109,138]. An integral part of 3D printing is bio-ink; therefore, the selection of the right bio-ink is crucial and must meet basic requirements such as gelation, shear thinning, printability, and viscoelasticity [139,140]. Agarose gel naturally possesses these properties and can meet printing requirements under certain conditions. Fan et al. [139] demonstrated that a hybrid matrigel-agarose hydrogel could be used as bio-ink for 3D printing, showing that the agarose component of the composite system could hold the 3D printed structure while the matrigel provided the necessary microenvironment for cell growth. Four-dimensional (4D) printing has recently attracted increasing research interest due to its unique properties. Four-dimensional printing uses special smart materials to print objects with more complex structures that can change shape over time in response to specific external stimuli [141]. Jinhua Guo prepared a 4D-printed agarose/laponite/polymerized acrylamide hydrogel using agarose as a substrate. The experimental results showed that the 4D gel could further deform and that the hydrogel changed shape over time through external treatment followed by cooling [142].

## 4. Materials and Methods

For a comprehensive description of the latest research on agarose extraction, agarose modification, and its applications in biomedical science, Science Direct (Elsevier B. V., Amsterdam, The Netherlands), Pubmed (US National Library of Medicine. Bethesda, MD, USA) and Web of Science (Clarivate PLC, London, UK) databases are all available for documentation searching. In the section on agarose extraction, the keywords “agarose, extraction” were used to search, and the results were filtered by title, abstract, and full text to select the best literature on agarose extraction from 1937 to 2023 for analysis. In the agarose modification and applications section, a search was conducted using the keywords “agarose, modification, biomedical applications”, and the results were filtered for title, abstract, full text, and articles published in top journals of the field within the last five years.

## 5. Conclusions

Agarose is a carbohydrate polymer with modifiable properties that are becoming increasingly attractive in biomedicine. The rigidity, porosity, electrical neutrality and hydrophilic properties of agarose hydrogel provide it with unique advantages for separation, and purification. Additionally, agarose contains numerous hydroxyl groups, which can be modified through chemical methods to obtain specific functional groups and the further derivation of different types of chromatographic separation media, which broadens its range of separation applications. In addition, its excellent biocompatibility makes agarose one of the most promising carriers for drug delivery systems. In particular, in targeted drug applications, agarose can be loaded into lesions for targeted drug delivery. Because of its inert structure, agarose has excellent biocompatibility. It is possible to modulate the structure of agarose to provide controlled permeability to air and nutrients and regulate cell adhesion. Therefore, agarose-based biomaterials could provide a living space environment for cells in a biomimetic environment.

## 6. Prospects and Challenges

Focusing on the biomedical field, agarose, and modified agarose show great potential for application because of their unique physicochemical properties. A great deal of research on agarose and its derivatives is still needed, however, to improve green extraction and modification. Firstly, the source of agarose needs to be considered, including differences in the composition or molecular weight of agarose when extracted from seaweeds grown in different environments; it is also necessary to select more appropriate extraction and modification techniques for agarose and choose eco-friendly solvents to improve yield and quality. Furthermore, predicting the function of agarose gels by modulating chemical modifications is a challenge because the relationship between the chemical structure and mechanical properties of agarose still needs to be discovered. 

Chromatography is one of the most efficient and gentle methods in separation and purification techniques. To achieve high-resolution purification, the particle size of microspheres and their homogeneity, as well as their fine pore structure, are very important factors. Agarose microspheres, commonly prepared by mechanical stirring and homogenous emulsification methods, are not uniform in size and require secondary sieving. Recently, membrane emulsification technology has been developed to prepare microspheres, which are characterized by uniform and controllable particle size, mild reaction conditions, simple operation, and green process [80]. However, several factors must be considered to obtain the desired microspheres, including the type of membrane, pH, solution viscosity, temperature, transmembrane pressure, continuous phase flow rate, and preparation efficiency. Therefore, this technique needs to be further explored.

Natural agarose is enriched with hydroxyl groups and is capable of forming hydrogen bonds with drugs and bioactive molecules for delivery. However, a limitation exists in employing raw agarose-based materials as drug delivery systems: the most cited regulations being a relatively high dissolution temperature, low degradation rate, low potential for the adsorption of hydrophobic drug molecules, and the slow adsorption/desorption of certain drugs [118]. Many studies have aimed at solving these problems through chemical modifications, yet the toxicity and biocompatibility of modified agarose still need to be addressed. Several studies have demonstrated the low cell adhesion, low in vitro cytotoxicity, and good biocompatibility of modified agarose [52,143,144]. However, most studies have remained in vitro, and the study of modified agarose-loaded drugs in animals still needs to be improved, which indicates that modified agarose-based drug delivery systems still have a long way to progress.

In the field of tissue engineering, agarose, and its derivatives are similarly lacking in animal testing. The exploration of biocompatibility, hemocompatibility, regenerative capacity, and toxicity in animals has, therefore, become a focus of research. At the same time, the natural brittleness of agarose has been a neglected aspect of research. Flexible agar hydrogels with self-healing capabilities have been developed [145]. With similar studies that can be drawn upon to address the in vivo compatibility of hydrogels, innovative agarose-based biomaterials are bound to have an increasing impact on biomedical applications.

## Data Availability

Not applicable.
